# An efficient alpha helix model and simulation framework for stationary electrostatic interaction force estimation

**DOI:** 10.1038/s41598-021-88369-3

**Published:** 2021-04-27

**Authors:** Guy G. Butcher, William S. Harwin, Chris I. Jones

**Affiliations:** School of Biological Sciences, University of Reading, Reading, England

**Keywords:** Computational models, Protein analysis, Protein folding, Software, Computational biophysics, Biophysics, Computational biology and bioinformatics

## Abstract

The alpha-helix coiled-coils within talin’s rod domain have mechanical and signalling functions through their unfolding and refolding dynamics. A better understanding of talin unfolding events and the forces that are involved should allow better prediction of talin signalling. To overcome the current limitations of force measuring in molecular dynamics simulations, a new simulation framework was developed which operated directly within the force domain. Along with a corresponding alpha-helix modelling method, the simulation framework was developed drawing on robotic kinematics to specifically target force interactions. Coordinate frames were used efficiently to compartmentalise the simulation structures and static analysis was applied to determine the propagation of forces and torques through the protein structure. The results of the electrostatic approximation using Coulomb’s law shows a simulated force interaction within the physiological relevant range of 5–40 pN for the rod sub-domains of talin. This covers the range of forces talin operates in and is 2–3 orders of magnitude closer to experimentally measured values than the compared all-atom and coarse-grained molecular dynamics. This targeted, force-based simulation is, therefore, able to produce more realistic forces values than previous simulation methods.

## Introduction

Platelets close vascular injury by aggregating, forming thrombi and then contracting^1,2^. A key element of this process are mechanosensitive proteins such as talin that provide a physical interface between the external environment of the platelet and the internal cytoskeleton and the contractile machinery, by sensing and adapting to forces exerted on the platelet^3–6^.

Talin’s rod domain contains 13 alpha-helix coiled-coils or ‘bundles’, which unfold if the tension applied to them exceeds a specific threshold. The surface of these alpha-helix bundles contain protein binding sites which are accessible while a bundle is folded, whilst inside the bundles cryptic binding sites exist which are only made accessible once a bundle has unfolded. The changes in accessibility of these binding sites and the subsequent binding or ejection of ligands, produces talin’s signalling response^7^. Thus, talin has a dual role, regulating force transmission within the platelet and regulating intra-cellular signalling in response to this force.

Twelve of talin’s 13 alpha-helix bundles unfold independently. These unfolding events occur between the tension ranges of 5–25 pN^8,9^. In platelets, talin experiences tensile forces within a physiologically relevant range up to 10 pN^10,11^. Within this limit only six alpha-helix bundles unfold^8^. The R3 bundle is the only bundle that has been proven to unfold within this reduced range^12^. Therefore, only one of the six bundles that unfold within talin’s normal physiological range of tension is mapped to its corresponding alpha-helix bundle. The remaining five unfolding events need to be connected to their alpha-helix bundles in order to fully understand talin’s role in platelet spreading, platelet contraction, focal adhesion formation and thrombus formation. Previous experimental work has explored the unfolding dynamics of talin’s mechanosensitive rod domain by stretching it using optical and magnetic tweezers^12–15^. However, limitations to this method, including high level of noise^16,17^ and low spatial and temporal resolution^18^, have made it challenging to identify the exact connection between alpha-helix unfolding events and their corresponding bundles.

Theoretical approaches have therefore been used to explore the dynamics of talin. Molecular Dynamics simulations, specifically all-atom models, offer a way to get a detailed perspective at the atomic level of the interactions between structures within the alpha-helix bundles^19,20^. Molecular Dynamics simulations, however, have the specific disadvantage that they can only simulate small portions of proteins over short time durations, due to the high computational costs involved in calculating the energies of bonded and non-bonded interactions between every atom in the simulation at each time-step^21^. Additionally, they are limited to short integration time-steps as to prevent the introduction of discretisation errors while sampling fluctuating atomic positions^22^. This high-level of detail combined with small integration time-steps and simulation duration can lead to the amplification of any inaccuracies in the force-field interaction model.

In addition, molecular dynamics simulations have difficulty calculating inter-protein forces and have only been able to modelled talin unfolding at supra-physiological forces. Previous all-atom and coarse-grained simulations of talin’s rod domain^20,21,23,24^, have stretched a section of talin’s rod domain by applying a constant velocity of $2\,\hbox {nm}\,\hbox {ns}^{-1}$ to one of the two terminal ends of the sub-domain. In these simulations, peak force values across an alpha-helix bundle can range from 400 to 1500 pN. These forces are one-to-two orders of magnitude larger than the peak forces measured experimentally using optical tweezers^8^. Commenting on this discrepancy Haining et al. state that the forces from these molecular dynamics simulation were relative and could not be directly compared with the results from physical experiments. As the force values from Molecular Dynamics simulations are not direcly comparable to previously recorded values, other methods needed to be explored.

At the other end of the theoretical spectrum, statistical models take known experimental information and reveal behavioural pattens^8^. However, the high-level view provided by statistical models does not provide the level of detail required to reveal information about the unfolding of specific alpha-helix bundles.

Both molecular dynamics simulations and statistical modelling have strengths but also specific disadvantages which make them a poor fit for solving the connection between unfolding event and specific talin bundles. Molecular dynamics is highly specific with high spatial and temporal resolution but at the sacrifice of ranges of spatial and temporal distances it can operate over at one time. The results are highly detailed but with limited larger context to situate them in. On the other hand, statistical analysis techniques can operate over large spatial and temporal ranges but with reduce resolution of both aspects, meaning it can lack the precision to determine the base mechanism from which a behaviour is generated.

To overcome the challenges of calculating the unfolding force thresholds of talin’s rod domains and determine the connections between the five remaining alpha-helix bundles and their corresponding unfolding events, we have developed a simulation framework using coordinate frame manipulation techniques from the field of robotic kinematics, along with methods to estimate static and dynamic forces and torques. Unlike Molecular Dynamics simulations that consider energy flow and conservation techniques, we designed the new simulation framework to operate directly within the force domain, eliminating the need to calculate an energy field gradient to establish a force.

The use of nested local coordinate frames linked with transforms is a new approach within the field of structural biology, based on techniques used in robotics. Combined with this, we have developed a simulation framework that operates within the force domain rather than the more common energy domain. This simulation framework does not compete with Molecular Dynamics. Instead, it attempts a problem that Molecular Dynamics was not designed to solve. Therefore, it is complementary to but wholly separate from Molecular Dynamics.

This paper will introduce the simulation framework targeted at the spatial and temporal ranges that talin operates within, along with the mathematical methods used to transform .pdb data, from the Protein Databank, into local coordinate frame based simulation objects. The simulation objects with their local coordinate frame will then be linked using coordinate frame transformations, which will be manipulated within the simulation using the mathematical framework of statics. The approximations used to construct these simulation objects will be evaluated. Finally, the results and computational cost of a static force simulation of alpha-helix bundle interactions forces from the talin protein’s rob sub-domains will be discussed.

## Methodology

### PDB data flow

The amino acid residues sequence of the talin rod domain was obtained from the SWISS-MODEL Repository^25^. A template sequence was selected for each of the rod sub-domains (R1–R13). Each sequence was selected by comparing template quality and sequence matching scores to other templates covering the same residue ranges and the template that matched the sequence most closely and with the highest quality scores was selected^26^. The final template for each alpha-helix bundle was downloaded from SWISS-MODEL as a .pdb file (PDBID: 6R9T, 2L7A, 2LQG, 5FZT, 4F7G, 2KVP, 3DYJ, 2JSW)^27–33,34,35^.

Additionally, SWISS-MODEL was then used to determine the positions of the alpha-helices within the template. This was done manually by visually matching a secondary structure overlay with the residues sequence indices for each helix. Indices for the starting and ending positions of each helix were stored. The .pdb files and their corresponding alpha-helix indices, were imported into MATLAB using the bioinformatics toolbox’s pbdread() function in order to parse the .pdb files into a usable data structure.

### Processing of MATLAB PDB objects

For the alpha-helix bundle being processed, each alpha-helix’s PDB data was grouped using the helix indices previously extracted from SWISS-MODEL. The data for each alpha-helix was then further separated by residue index such that each amino acid’s data in the amino acid chain was grouped. The data that contained the positions of all of the amino acid’s atoms was then separated into backbone atoms and sidechain atoms. This was done by selecting all the atoms whose “Atom Name” was either “O”, “N”, “C” or “$\text {C}\alpha$” as the backbone atoms. The sidechain atoms were therefore all the remaining atoms.

### Generation of helix object

#### Backbone

A local coordinate frame was associated with each alpha-helix using the following method.

Each amino-acid residue’s backbone position was approximated to a single point, the backbone position. The position of the $\text {C}\alpha$ atom in the backbone structure was selected as the backbone position for two reasons. It was placed centrally in the residue backbone structure and the residues sidechain structure is directly connected to the backbone through this atom. We defined a matrix ${\mathbf {M}}$ as $${\mathbf {M}} = \begin{bmatrix}\underline{b_1}\dots \underline{b_i}\dots \underline{b_n}\end{bmatrix}^{\intercal }$$ where the vector $\underline{b_i}$ is a column vector that contains the triplet of backbone positions in .pdb coordinate space , for each residue in the alpha-helix. The vector of positions $\underline{b}_{i}$ contains the backbone position, in .pdb coordinate space, for each residue in the alpha-helix. The symbol *i* denotes the *i*th amino-acid where $i = 1,\dots ,n$ and *n* is the total number of amino-acid residues in the alpha-helix.

For use in future calculations, the mean position of all the backbone positions was determined. The position vector $\underline{b_c}$ corresponds to the centroid of backbone positions of the alpha-helix.

1 $$\begin{aligned} \underline{b}_{c} = \frac{1}{n} \sum ^{n}_{i=1}{\underline{b}_{i}} \end{aligned}$$

An axis along the backbone, corresponding approximately to the alpha-helix axis was determined using principal component analysis (PCA). The PCA was calculated using single value decomposition (SVD) of the zero mean $\text {C}\alpha$ backbone positions. The variance–covariance matrix ${\mathbf {S}}$ was formed as shown below.

2 $$\begin{aligned} {\mathbf {B}}= \,& {} \begin{bmatrix} \left( \underline{b_1} - \underline{b_c} \right) \dots \left( \underline{b_i} - \underline{b_c} \right) \dots \left( \underline{b_n} - \underline{b_c} \right) \end{bmatrix} \nonumber \\= \,& {} {\mathbf {M}} - \underline{r}\underline{b_c} \end{aligned}$$

3 $$\begin{aligned} {\mathbf {S}}= \,& {} \frac{{\mathbf {B}}^{\intercal }{\mathbf {B}}}{n-1} \end{aligned}$$

${\mathbf {B}}$ is a *n*-by-3 matrix of alpha-helix backbone ($\text {C}\alpha$) positions with a zero-mean which was created by element-wise subtraction of $\underline{b_c}$ from ${\mathbf {M}}$, this can also be constructed by setting temporary vector $\underline{r}$ to a row vector containing 1 in all *n* columns. ${\mathbf {S}}$ is thus simply the variance–covariance matrix of ${\mathbf {B}}$. Matrix ${\mathbf {S}}$ was then decomposed in order to obtain the principal axes from the left matrix of singular vectors that corresponds to the largest singular value. ${\mathbf {U}},\varvec{\Sigma }$ and ${\mathbf {V}}$ are the left matrix of singular vectors, diagonals of the singular values, and right matrix of singular vectors of ${\mathbf {S}}$ respectively.

4 $$\begin{aligned} {\mathbf {U}}\varvec{\Sigma }{\mathbf {V}}^{\intercal } = {\mathbf {S}} \end{aligned}$$

The vector approximating the direction of the alpha-helix backbone structure was estimated from the first left singular vector which corresponds to the largest singular value. $\underline{{\hat{d}}}$ is the first principle axis through the positions in ${\mathbf {B}}$, in other words the direction of the central axis through the alpha-helix backbone, $u_{*,1}$ specifies the vector forming the first column of elements from ${\mathbf {U}}$. Therefore, $\underline{{\hat{d}}}$ is the first singular vector of ${\mathbf {S}}$ and all the other singular vectors could be ignored.

5 $$\begin{aligned} \underline{{\hat{d}}} = \underline{u}_{*,1} \end{aligned}$$

The line through the alpha-helix backbone structure, nominally the backbone axis, was defined by the two positions $\underline{p}_{s}$ and $\underline{p}_{e}$ which are the *start* and *end* axis positions. These positions were calculated using the parametric form of the line equation as shown in Eq. (6).

6 $$\begin{aligned} \begin{aligned} \underline{p}_{s}&= \underline{b}_{\text {c}} + \underline{{\hat{d}}} \, \mathopen | \underline{b}_{\text {1}} - \underline{b}_{\text {c}} \mathclose | \\ \underline{p}_{e}&= \underline{b}_{\text {c}} + \underline{{\hat{d}}} \, \mathopen | \underline{b}_{\text {n}} - \underline{b}_{\text {c}} \mathclose | \end{aligned} \end{aligned}$$

$\underline{p}_s$ and $\underline{p}_e$ are the start and end positions for the alpha-helix backbone cylinder approximation respectively, $\underline{b}_{\text {1}}$ and $\underline{b}_{\text {n}}$ are the first and last position vector elements of the alpha-helix backbone positions ${\mathbf {B}}$. The positions $\underline{p}_s$, $\underline{b}_c$ and $\underline{p}_e$ are collinear.

The approximated size or radius of the alpha-helix can now be estimated using Eqs. (7) and (8). The shortest distance between each backbone position and the backbone axis line was calculated. This equation computed the position along the backbone axis line that resulted in a perpendicular line from the backbone axis line to the backbone position.

7 $$\begin{aligned} \begin{aligned} \underline{p}_{a}(t)&= \underline{p}_{s} + t (\underline{p}_{e} - \underline{p}_{s}) \\ t(\underline{p}_{x})&= \underline{{\hat{d}}} \, \frac{(\underline{p}_{s} - \underline{p}_{x})}{\mathopen | \underline{p}_{e} - \underline{p}_{s} \mathclose |} \\ \underline{{\hat{d}}}&= \frac{(\underline{p}_{e} - \underline{p}_{s})}{\mathopen | \underline{p}_{e} - \underline{p}_{s} \mathclose |} \end{aligned} \end{aligned}$$

$\underline{p}_{a}$ is the position calculated along the line defined by positions $\underline{p}_{s}$ and $\underline{p}_{e}$, *t* specifies the distance along this line. $\underline{{\hat{d}}}$ is the unit vector specifying the direction from point $\underline{p}_{s}$ to point $\underline{p}_{e}$

8 $$\begin{aligned} l_{i} = \left\Vert \underline{b}_{i} - \underline{p}_{a} \right\Vert = (\underline{b}_{i} - \underline{p}_{a})^{\intercal }(\underline{b}_{i} - \underline{p}_{a}) \end{aligned}$$

$p_a$ is the result of Eq. (7) where $t(\underline{b}_{i})$, $l_{i}$ is the Euclidean distance between $\underline{p}_{a}$ and the $i^{\text {th}}$ backbone position.

#### Sidechain

To approximate each sidechain as a single position, the centre of atoms was determined by taking the mean value of all the positions of the atoms in each sidechain. For each sidechain on the alpha-helix, $\underline{p}_{\text {SC},i,j}$ was set as the 3D position vector of a sidechain atom. Where *i* specifies the specific sidechain in the alpha-helix where $i = 1,\dots ,n$, and *j* specifies a sidechain atom where $j = 1,\dots ,m$. The value *n* is the total number of backbone or sidechain positions and *m* is the total number of sidechain atoms in the $i^{\text {th}}$ sidechain. Equation 9 was used to calculate the approximate position of the sidechain. $\underline{s}_{i}$ is the approximated position of the $i^{\text {th}}$ sidechain. The *i* value links the *i*th sidechain to the *i*th backbone position.

9 $$\begin{aligned} \underline{s}_{i} = \frac{1}{m} \sum ^{m}_{j=1}{\underline{p}_{\text {SC},i,j}} \end{aligned}$$

The sidechains were then approximated as spheres whose centre was at the mean sidechain atom position, $\underline{s}_{i}$. The radius of these approximation spheres was estimated from the Euclidean distance between the approximated sidechain position and each of the sidechain atoms positions. The mean values of these distances was the taken as the radius of the sidechain sphere, $\underline{r}_{i}$, as shown in Eq. (10). Amino acid sidechain are generally planar in configuration. However, they posses rotation freedom around the covalent bond which links the sidechain to it’s backbone’s carbon-alpha atom. Hence a sphere approximation is appropriate. The approximated sphere radius is calculated as show in Eq. (10). $\underline{r}_{i}$ is the approximated size of the $i^{\text {th}}$ sidechain as a radius of an approximated sphere.

10 $$\begin{aligned} \underline{r}_{i} = \frac{1}{m}\sum ^{m}_{j = 1}{\left\Vert \underline{p}_{\text {SC}_{i,j}} - \underline{s}_{i}\right\Vert } \end{aligned}$$

### Application of local coordinate frames

All positional coordinates so far have been within a global PDB coordinate frame. This frame was the coordinate space within the .pdb file. Coordinate transforms were used to allow individual alpha-helices to be considered as moving with respect to each other. Coordinate transforms were used and in general these calculations were done as homogeneous transforms^36^. The kinematics techniques from robotics allows the positional data to be considered in different coordinate frames simply by multiplying the appropriate matrices together. Similar techniques are used in computer graphics, but this paper follows the conventions used in robotics as this eases subsequent calculations of force and torque.

Although there are many methods to specify the location and orientation of a coordinate frame, there is much flexibility available from the homogenous transform representation given in Eq. (11).

11 $$\begin{aligned} {^{\alpha {\text {H}}\#}_{\text {PDB}}}{\mathbf {T}} = ({\mathbf {R}},\underline{p_0}) = \begin{bmatrix} &{}&{}&{} \\ &{}{\mathbf {R}} &{} &{} \underline{p_0} \\ &{}&{}&{} \\ 0 &{} 0 &{} 0 &{} 1 \end{bmatrix} = \begin{bmatrix} &{}&{}&{} \\ \underline{{\hat{x}}} &{} \underline{{\hat{y}}} &{} \underline{{\hat{z}}} &{} \underline{p_0} \\ &{}&{}&{} \\ 0 &{} 0 &{} 0 &{} 1 \end{bmatrix} \end{aligned}$$

Thus Eq. (11) specifies the the coordinate transform from PDB space ($\text {PDB}$) to a local alpha-helix space ($\alpha {\text {H}} \#$) can be specified as a homogeneous transform, where ${\mathbf {R}}$ is the 3-by-3 rotation matrix, $\underline{p_0}$ is the 3D translation vector of the origin and $\underline{{\hat{x}}}$, $\underline{{\hat{y}}}$ and $\underline{{\hat{z}}}$ are the basis vectors that define the direction of the three axes for the $\alpha {\text {H}} \#$ space. The coordinate transform could also be applied through a two-step process of a rotation and then a translation using the ${\mathbf {R}}$ and $\underline{p_0}$ components separately, by an appropriate partitioning of the transform matrix.

#### Defining a transform for each alpha-helix local coordinate frame

The axes of the local coordinate frame can be defined by the three unit vectors as follows.

12 $$\begin{aligned} \begin{aligned} \underline{{\hat{x}}}&= \underline{{\hat{d}}} = \frac{(\underline{p}_{e} - \underline{p}_{s})}{\mathopen | \underline{p}_{e} - \underline{p}_{s} \mathclose |} \\ \underline{{\hat{y}}}&= \frac{(\underline{s}_{1} - \underline{p}_{a})}{\mathopen | \underline{s}_{1} - \underline{p}_{a} \mathclose |} \\ \underline{{\hat{z}}}&= \underline{{\hat{x}}} \times \underline{{\hat{y}}} \end{aligned} \end{aligned}$$

The position vector $\underline{s}_{1}$ is for the first non-glycine sidechain on the alpha-helix, $\underline{p}_{a}$ is the result of Eq. (7). The rotational sub-matrix and origin position vector are then as follows.

13 $$\begin{aligned} \begin{aligned} {\mathbf {R}}&= [\underline{{\hat{x}}},\underline{{\hat{y}}},\underline{{\hat{z}}}] \\ \underline{P}&= \underline{p}_{0} \end{aligned} \end{aligned}$$

Equation 12 can be considered as a rotational transformation ${\mathbf {R}}$ where along with $\underline{P}$ defines a coordinate transform from PDB space to $\upalpha$ helix space ($\upalpha$ H# where # is an integer value). $\upalpha$ Helix space is a local coordinate frame define around each alpha-helix. The centre of the frame is at one end of the alpha-helix, nominally known as the Start, $\underline{p}_{s}$. With the X-Axis aligned along the axis of the alpha-helix and the Y-Axis perpendicular to the X-Axis and pointing towards the first significant sidechain position. A significant sidechain is any non-glycine sidechain.

### Defining transformations for a two-helix simulation

A new coordinate frame, SIM, was defined to contain the active simulation. The first alpha-helix’s coordinate frame was placed within the SIM frame and the coordinate transform from $\upalpha$ H1 (the local coordinate frame containing the first alpha-helix in the current alpha-helix bundle) to SIM was defined and is shown in Eq. (14). A coordinate frame can be used to define the relationship between any coordinate frame and any other coordinate frame. The transform was set as in identity matrix in order to align the axes of $\upalpha$ H1 and SIM completely. The forward superscript annotation of ${\mathbf {T}}$ comes from Craig’s notation^36^.

14 $$\begin{aligned} ^{\text {SIM}}_{\alpha \text {H}1}{\mathbf {T}}= \,& {} \mathbf {I_{4\times 4}}\end{aligned}$$

15 $$\begin{aligned} ^{\text {SIM}}_{\alpha \text {H}2}{\mathbf {T}}= \,& {} {}^{\text {SIM}}_{\alpha \text {H}1}{\mathbf {T}} \ {}^{\alpha \text {H}1}_{LNK}{\mathbf {T}} \ {}^{LNK}_{\alpha \text {H}2}{\mathbf {T}} \nonumber \\ ^{\text {SIM}}_{\alpha \text {H}2}{\mathbf {T}}= \,& {} {}^{\text {SIM}}_{LNK}{\mathbf {T}} \ {}^{LNK}_{\alpha \text {H}2}{\mathbf {T}} \end{aligned}$$

The second alpha-helix’s frame ($\upalpha$ H2) was placed in the first alpha-helix’s frame ($\upalpha$ H1) and the intermediate linker transform, to and from the LNK frame, defines the offset from the end of the first alpha-helix, through the unstructured linker domain to the starting position of the second alpha-helix. This is shown in the first line of Eq. (15). As the the $\alpha \text {H}1$ frame was aligned to the SIM frame, a simplified version is shown in the second line of Eq. (15).

### Implementation of the static force simulation

**Figure 1:**
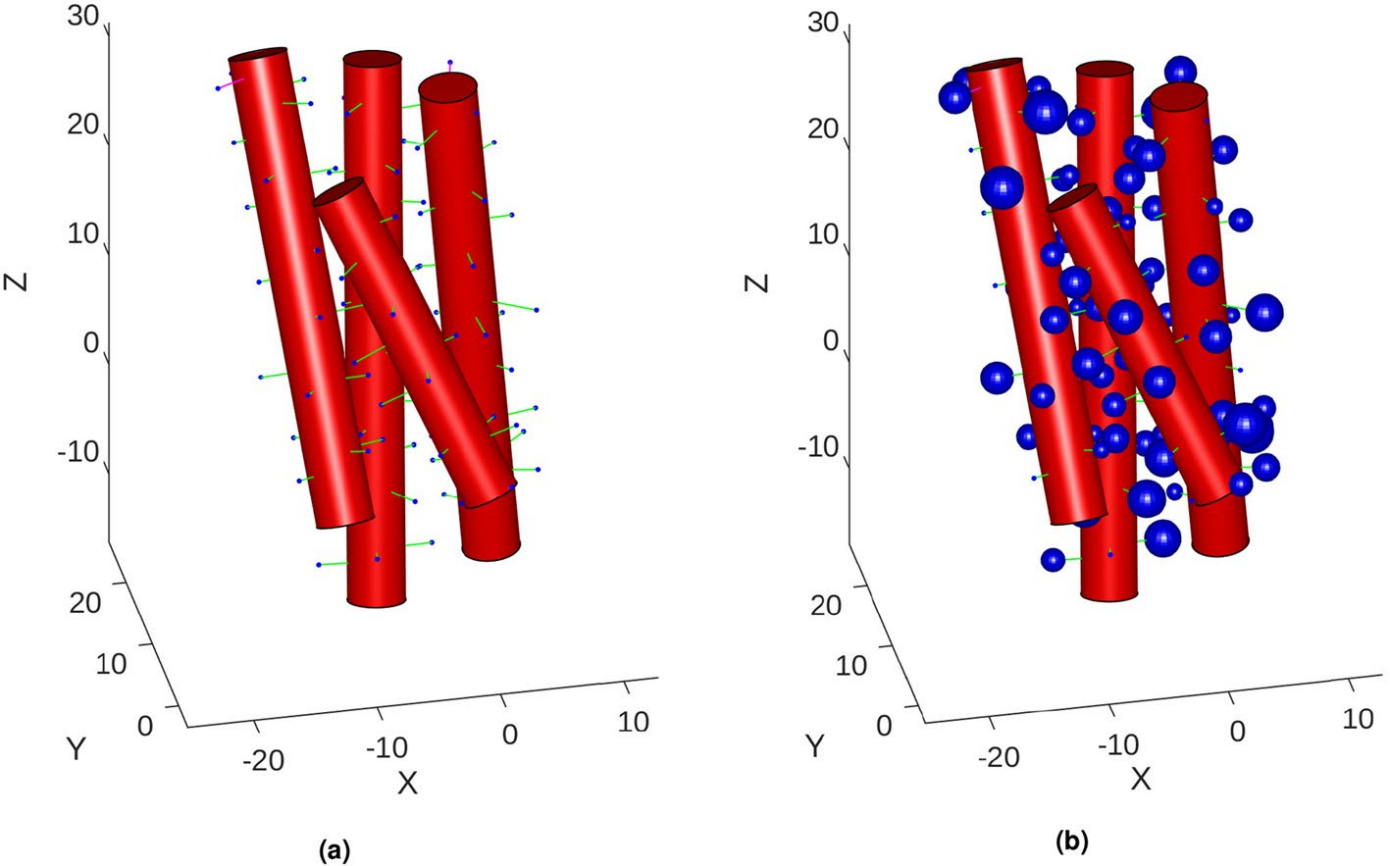
Visualisation of the R8 sub-domain’s alpha-helix objects within the simulation. The relative positioning and rotations were determined from the source .pdb file. Each blue dot (**a**) or blue sphere (**b**) represents a single sidechain position, the size of the blue spheres represent the approximated size of the sidechain and the green lines show the perpendicular direction to the backbone axis. The units of distance are Angstroms. For 3D Visualisation, see supplementary material. (MATLAB, 2020b, https://www.mathworks.com/).

To calculate the total interacting forces between all helices in an alpha-helix bundle, each helix was positioned in the simulation coordinate frame or in other words a global or base frame. Each helix was then transformed to be aligned to the same position and orientation which it inhabited in PDB coordinate space. Therefore, the relative positions and orientations of each helix towards each other was that same as that recorded in the .pdb file. Figure 1 contains a visual output from the static force simulation. It shows the four alpha-helices of the R8 sub-domain in the .pdb file configuration.

The electrostatic interactions between the helices were calculated using Coulomb’s Law, Eq. (16), where $k_e$ was Coulomb’s Constant or $\frac{1}{4\pi \epsilon _0}$, $q_1$ and $q_2$ were the point charges for which the force is being calculated between and $r^2$ was the squared distance between the two point charges.. The calculation was carried out on each non-repeating combination of pairs of alpha-helices. Within the pair, Coulomb’s Law was applied to each non-repeating pair of sidechain charges across the two helices. For the purpose of maintaining simple and unambiguous charges, the charge values were limited to integer values of $-1$, 0 and $+1$ and the protonation of each sidechain was taken at the physiological pH value of 7.2. All non-neutral sidechains were set as the closest non-zero charge value.

16 $$\begin{aligned} F = k_e\frac{q_1 q_2}{r^2} \end{aligned}$$

## Results

### Validation of modelling approximations

During the development of the alpha-helix model, a number of assumptions and simplifications were applied to their structure. This enabled a reduction in the complexity of the alpha-helix model which made them easier and faster to manipulate.

#### Backbone approximation

**Figure 2:**
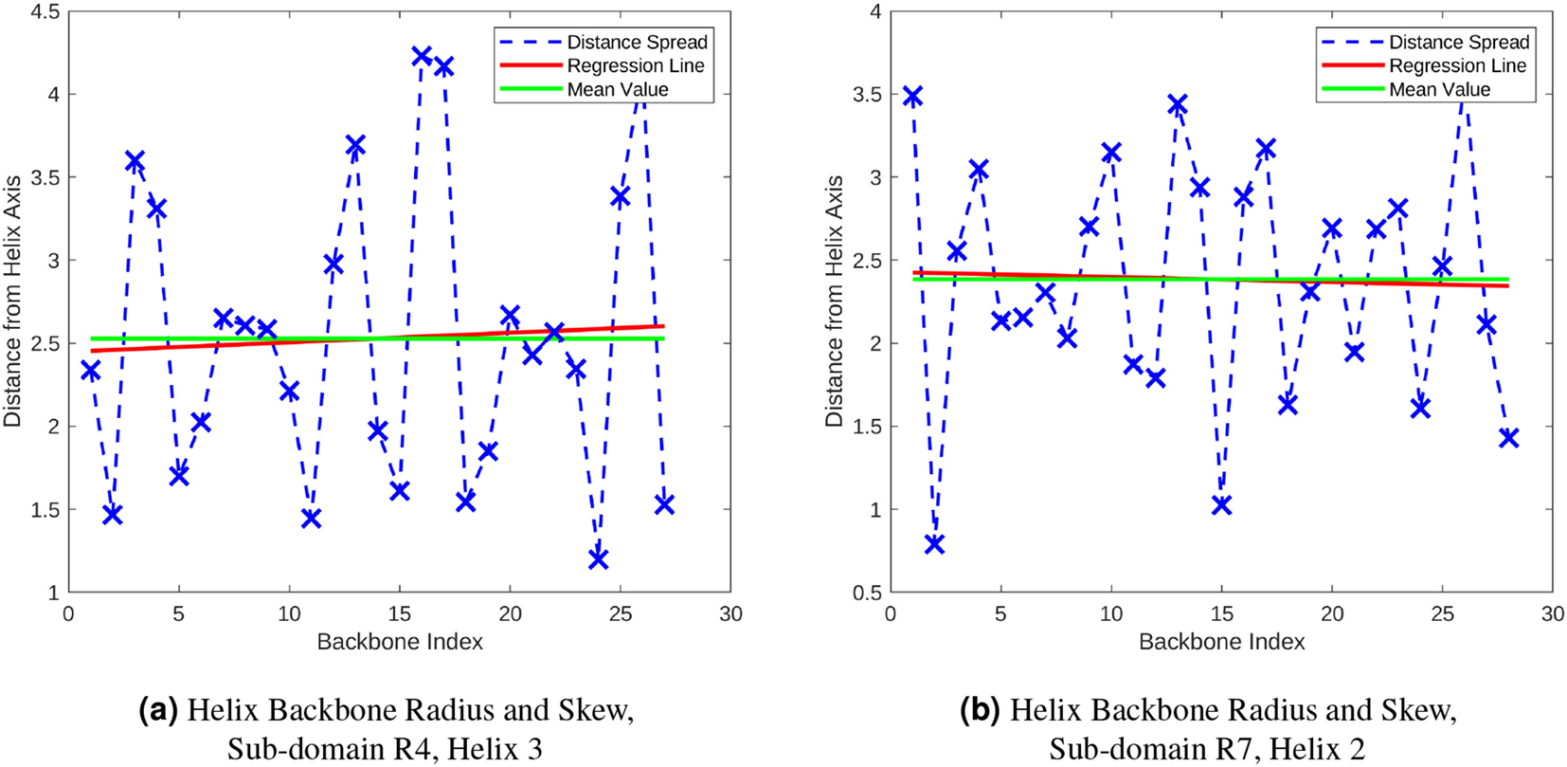
(**a**,**b**) The distances between each of the original backbone (C$\upalpha$) positions and the centre axis of the helix are shows. These plots allow the changing width of the alpha-helix backbone to be visualised. The green line represents the mean distance to the alpha-helix backbone from the centre axis. This is the value used for the width of the alpha-helix within our model. The red line shows the skew in the distance data along the length of the helix. A large difference in angle between the skew line and the mean line indicates that a helix’s width changes along is length. (MATLAB, 2020b, https://www.mathworks.com/).

**Figure 3:**
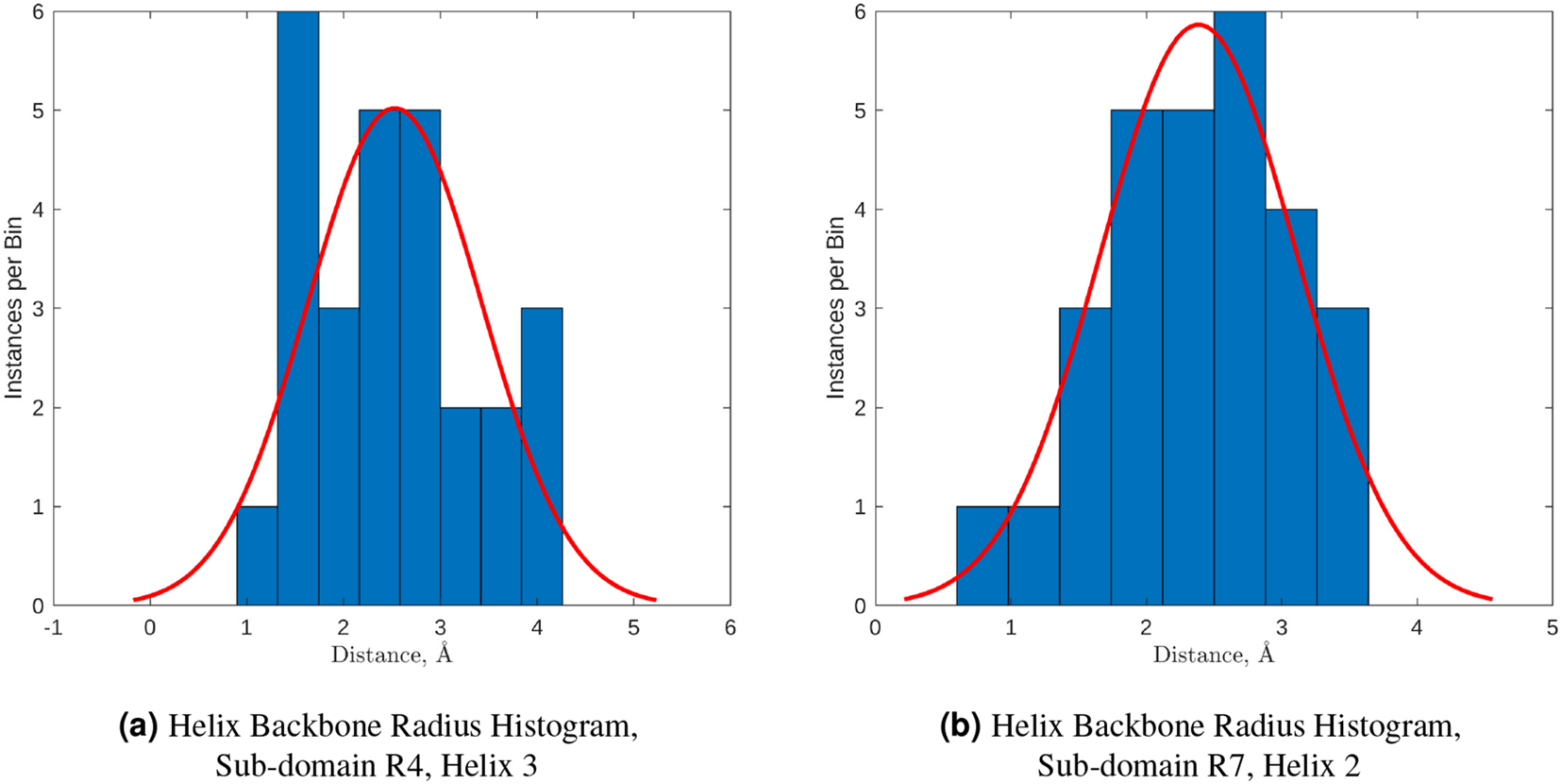
(**a**,**b**) The collections of distances were appropriately binned, plotted as histograms and a normal distribution was fitted. (The same data was used as in Fig. 2b.) This indicates that the distribution of the distances is not biased and that the mean value was a reasonable choice for the backbone radius. (MATLAB, 2020b, https://www.mathworks.com/).

The alpha-helix backbone had two assumptions applied to it that resulted in the approximated form in the model. (1) An alpha-helix is a straight, rigid structure, and (2) the size of the rigid alpha-helix is constant along its length. From X-ray crystallography, it is known that alpha-helices in coiled-coil domains can be curved and twisted. However, the element of the alpha-helix structure that is used directly in the force calculations are the sidechain’s electrostatic atoms. Therefore, as long as the relative positions between the approximated backbone structure and the sidechains were maintained, these assumptions can be made use of. The second approximation was used to remove the amino-acid backbone positions and replace them with an axis through their core that represented the direction and length of the alpha-helix. The width of the alpha-helix was then approximated as the mean distance between axis and each of the position that original held an amino-acid backbone. To test this approximation with its original assumption, amino-acid backbone positions with respect the axis were collected for each alpha-helix within talin’s rod domain and analysed. The comparison between original position data, the mean axis and the skew (the angle of the primary axis through the data) of the original positions were compared and scatter-plotted, Fig. 2. The orthogonal distance between the original positions and the axis were plotted as a histogram with a normal distribution fitted, Fig. 3.

**Figure 4:**
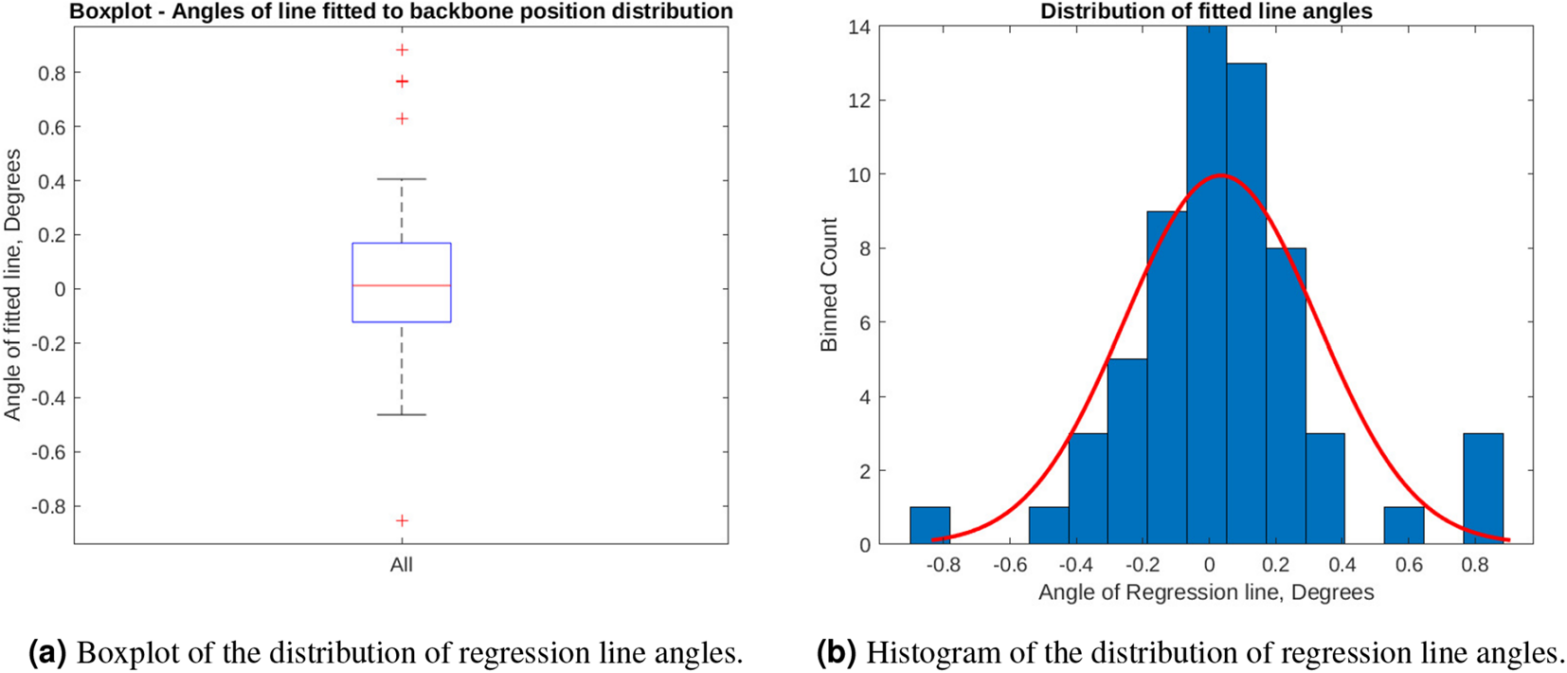
(**a**,**b**) show that the distribution of differences in angles between the regression lines, which represent the skew of the width of the alpha-helix backbone structure, and the average line, which represents approximated width of the alpha-helix. The difference in angles have a median value of 0.0122 degrees, an mean of 0.0350 degrees and a standard deviation of 0.2906 degrees. (**b**) also shows the the distribution is more specific than a normal distribution and the majority of the angles are near zero. (MATLAB, 2020b, https://www.mathworks.com/).

To further check these results, the distribution of the difference between the regression line and mean value were compared across all helices. The data in Fig. 4 shows that for the majority of the alpha-helices within talin’s rod domain, the width of the helix may be considered constant as all of the alpha-helices vary less than 1 degree from zero with a mean of 0.0350 and a standard deviation of 0.2906 degrees. It also shows that the calculated axis which was used to represent the alpha-helix backbone, closely resembles that of the core of the alpha-helices even through it is straight. Having a reasonable approximation of the alpha-helix size allowed us to render useful graphical representations, and will be needed during the future versions of the simulation framework for collision detection via bounding boxes.

#### Sidechain approximation

The sidechain’s of the amino-acids that comprise each alpha-helix, also were also approximated under an assumption. That each sidechain’s position could be approximated to the average position of all of its constituent atoms. This is consistent with the Levitt and Warshel model of sidechain modelling. Models with additional detail could have been used such as CABS or UNRES. However, this would have doubled or tripled the complexity of the modelling process by either defining a directionality of the sidechain representation or adding an intermediate joint between the sidechain and the backbone representation. Alternatively, simpler models could have been employed such as SICHO but this would have reduced the sidechains position to that of the core of the amino-acid^37^.

**Figure 5:**
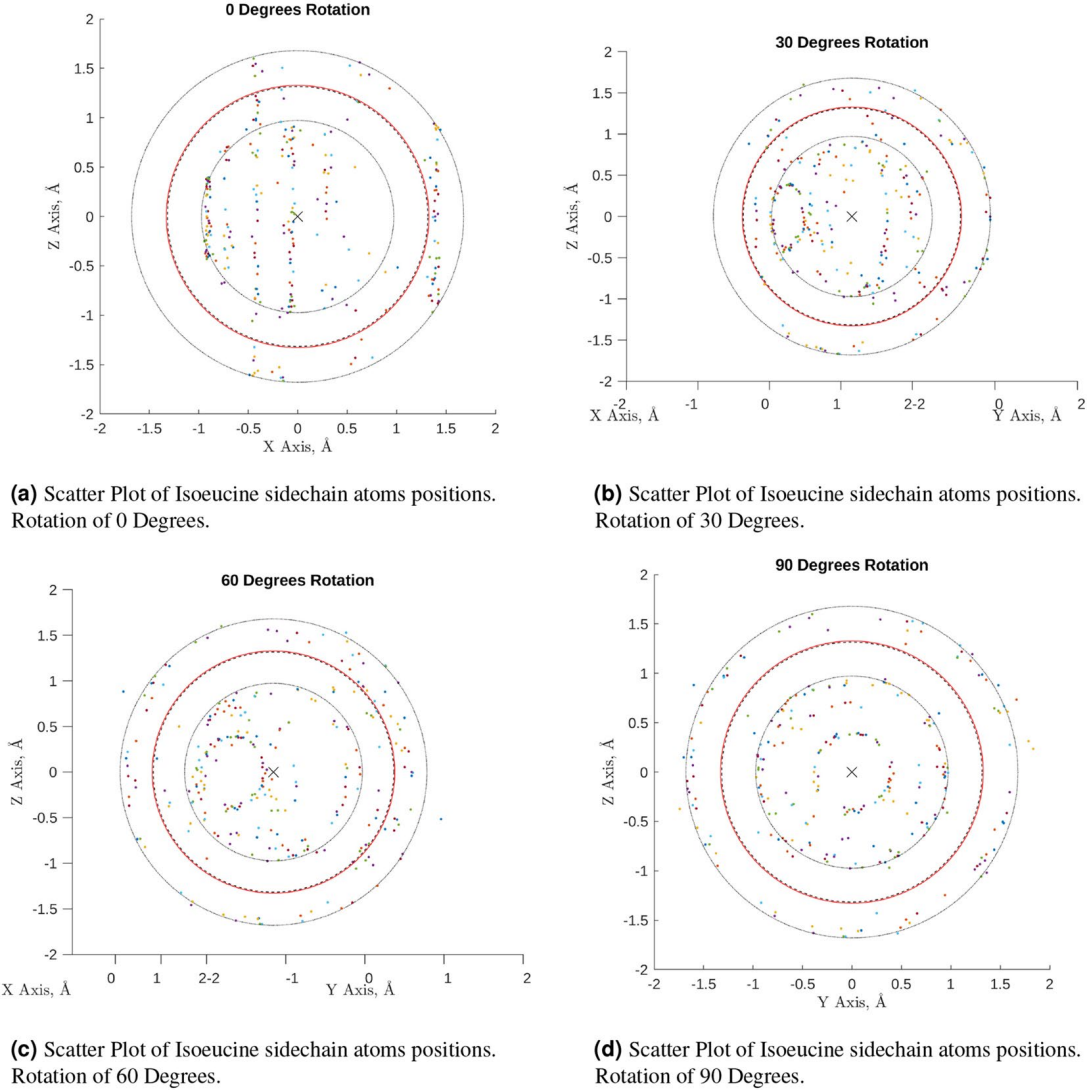
(**a**–**d**) The above scatter plots show the overlaid positions of the sidechain atoms from each alpha-helix in talin’s rod domain. Each plot shows a subsequent rotation around the Z axis of the view of the figure. The positions were first normalised with respect to their C$\upalpha$ atom position and then their orientations were aligned using the line between the average sidechain atom position and the C$\upalpha$ atom position. The red circle represents the mean Euclidean distance from the centre of the collection of sidechain positions to each individual atoms position. The dashed line represents the median distance, the inner dotted line represents the first inter-quartile and the outer dotted line represents the third inter quartile. (MATLAB, 2020b, https://www.mathworks.com/).

This approximation was checked for each type of amino-acid sidechain. The positions of every atom in the sidechain were plotted, overlaid with the same positions for each instance of that type of sidechain, from all of the alpha-helices within talin’s rod domain. The relative orientations of each sidechain were not normalised before plotting. This allowed the average shape of each sidechain to be viewed, irrespective of their orientation. Figure 5, shows two of these collections of sidechain atom positions.

### Static force estimations of alpha helix bundles

Each alpha-helix bundle was simulated as a separate instance. The origin of the simulation space was set at the N-terminus of the first alpha-helix within the four or five chain sub-domain. The orientation of the first alpha-helix and each subsequent helix was determined from their conformation in .pdb that their structure was extracted from. Therefore, the orientation and relative placement of each alpha-helix was identical to the PDB structure.

Coulombs law was used to determine the electrostatic forces between each possible, non-repeating pair of sidechains between each non-repeating pair of alpha-helices. The electrostatic interactions were combine into a static force value that represents of overall tendency of the bundles alpha-helices to be attracted or repulsed from each other. These static force values for each alpha-helix bundle are presented in Table 1.

**Table 1:** The calculated electrostatic force values between the alpha-helices for each alpha-helix coiled-coiled sub-domain within talin’s rod domain.

Bundle	Force, pN
R1	− 30.9
R2	18.6
R3	− 8.90
R4	22.0
R5	− 40.3
R6	− 20.5
R7	− 7.28
R8	12.2
R9	− 5.27
R10	− 24.5
R11	− 29.3
R12	− 11.0
R13	− 43.6

All alpha-helices within each sub-domain were statically positioned. Negative force values represent attractive interaction and positive force values represent repulsive interactions. Therefore, the three outliers were R2, R4 and R8 within unexpectedly repulsive interactions.

In terms of absolute force values, the simulation force results range from 5.26 pN to 43.6 pN, which fully includes the ranges of unfolding force events previously recorded^8,14^. The force values were also at the order of magnitude expected, $10^{-12}$ to $10^{-13}\,\hbox {N}$. This is an improvement on the accuracy of computational results when compared to all-atom and coarse-grained molecular dynamics approaches, whose results range in orders of magnitude from $10^{-9}$ to $10^{-12}\,\hbox {N}$^21,23^. The improved range of interaction force values show that this first iteration of this simulation method is making progress within this area, even while only looking at a single non-bonded interaction. Thus, this is an improvement in the specific area of interaction force estimation when compared to all-atom and coarse-grain molecular dynamics simulation on talin-1’1 rod domain.

## Discussion

Understanding the forces involved in the unfolding of the rod-domain of talin is critical if we are to understand how platelets regulate the forces they are subjected to, and convert external physical stimuli into an intra-cellular signalling response. The techniques described would aslo extend to understanding dynamics limitations of other cellular structures. To overcome the current challenges of calculating the unfolding force thresholds of talin’s rod domains, a new simulation framework was developed using coordinate-frames attached to substructures (in this case the alpha-helix) in order to simulate interaction force between them. The first iteration of a purpose-built simulation framework was presented. This framework operated directly within the force domain, based on principles used in the field of robotics, and made use of an approximated model alpha-helices and their sidechains for computational efficiency. The simulation framework was specifically designed to operate within the spatial and temporal ranges within which talin unfolding occurs $\approx 5\,\hbox {pN}$ to $\approx 25\,\hbox {pN}$ and $\approx 100\,\hbox {nm}$ to $\approx 800\,\hbox {nm}$^8^. Although we present the first iteration of this model containing only electrostatic forces, the results of the static force simulations show the majority of the bundles modelled developed attractive force within the expected physiological relevant ranges for talin. These forces are closer to the expected unfolding forces of talin, that were determine via energy-based simulations such as molecular dynamics, by one-to-three orders of magnitude when compared to values generated previously through traditional and coarse-grained molecular dynamics simulation.

Simulations such as molecular dynamics must account for a number of inter- and intra-molecular interactions. As our simulation focused on intra-alpha-helix interactions and the models used approximate away internal structures, we started by examining the non-bonded interactions between alpha-helices. The non-bonded interactions primarily consist of long-range electrostatic forces, hydrophobic interactions and van der Waal forces. The two most prominent forces, by magnitude, in the above list were electrostatic and hydrophobic^38^. Opinions differ over whether one is more prominent or more powerful than the other. Therefore, to test the principle of the simulation framework the simplest to implement, electrostatic force which can be calculated as simple point-to-point interactions under Newtonian physics, was selected as the first form of interaction to simulation.

Hydrophobic interactions are not a direct force interaction but a result of a system tending towards a lower energy state. This is achieved by minimising the amount of hydrophobic amino-acid residue sidechains protruding into the surrounding aqueous environment. The hydrophobic sidechains interrupt the water molecules’ natural lattice matrix, thereby reducing the number of potential configurations a single water molecule can take at any one time. This decreases the randomness or disorder of the water’s structure and therefore increases the systems entropy. By hiding the hydrophobic sidechain inside the core of the proteins structure, the water molecules are able to position themselves in a larger number of configurations and thus the system is more disordered and has lower entropy. Additional work is needed to establish a useful was of calculating hydrophobic interactions such that these can be combined or compared with the Coulomb based method previously outlined.

Despite its simplification the majority of the bundles in our model showed attractive force in the correct order of magnitude, indicating the potential utility of this approach. Three, however, showed repulsive forces. The repulsive force values the three alpha-helix bundles (R2, R4 & R8), are in contrast to the current evidence for the unfolding events for the talin rod domain and therefore require further investigation. The first reason why these values might be present, is the lack of additional intra-molecular forces in the simulation calculations. The non-covalent electrostatic force is one of the highest magnitude forces that interact in this scale of distance and time. The other most impactful interaction at these scales is the hydrophobic interaction. If this was included, it can be assumed the effect would be an increase in the attractive force values for all bundles. As the overall energy level of the system would tend to ‘hiding’ the hydrophobic core of the alpha-helix bundles. By including hydrophobic interaction and/or van der Waal interactions the accuracy of the results would undoubtedly improve, and these are planned future additions.

The simulation and interaction results are static within the current iteration of the framework. Coordinate frames systems and the field of manipulator dynamics contains a collection of equations that enable dynamic forces of a moving system to be calculated over time. This could be added to the simulation framework to enable the full unfolding and refolding dynamics of the alpha-helix bundles to be recorded and explored. In addition, future iterations will make provision for alpha-helices that are curved and bend while within a folded bundle conformation. The introduction of flexibility into the alpha-helix model would enhance to dynamic simulation.

Similarly, the current simulation does not include the linker regions between alpha-helices. These unstructured polypeptide chains range between one to tens of amino-acids in length. Some linker regions are beta-turns, some have hydrogen bonds which link the beginning and end sections of the linker together while other are completely unstructured with no obvious characteristics. By taking into account these regions, especially the ones with restricted conformations due to bonds or short structures, could add important data that help explains the missing attractive forces some bundles within the current model.

In summary, we have developed a simulation framework, based on sub-elements each with their own coordinate-frame. This technique draws on principles developed for robotic kinematics and dynamics and allows us to model the unfolding forces in the alpha-helix coiled-coils of the rod-domain of talin. The first iteration of this model presented here relies solely on electrostatic force, yet produced results that are significantly closer to the expected unfolding forces of talin, determine by experimentation, than previous coarse-grained molecular dynamics simulations of talin unfolding. These initial results demonstrate the utility of this novel approach which will be refined in future iterations and applied to other mechanosensitive proteins.

Additions to this research direction may improved our understanding of the link between amino-acid sequence and structure, specifically helical structures. Which could result in further reverse engineering of proteins structures. This in turn would lead to better modelling for protein secondary structure prediction from base sequencing.

## Conclusion

This research demonstrates a robust methodology to generate simple models of individual alpha-helices that can then be used within a simulation to determine intra-molecular forces inside alpha-helix bundles. The simulation framework was explicitly developed to improve the understanding of the forces involved in the unfolding alpha-helix domains. It makes use of methodologies from the field of robotics such as coordinate frame systems and statics. The use of nested local coordinate frames linked with coordinate transforms to contain simulatable objects is a new approach within structural biology. Implementing the simulation framework directly within the force domain rather than in the traditional energy domain is also novel. The static force data generated by this model are closer in value to experimental results of the forces involved in talin unfolding by one to three orders of magnitude compared to similar simulation attempts using coarse-grained molecular dynamics methods. This demonstrates the potential utility of this approach over traditional all-atom and coarse-grained molecular dynamics simulations in this specific situation. This work establishes a new approach to understanding cellular structures that could allow better understanding of the processes and dynamics of force based molecular signalling. Future work is needed to include non-bonded interactions, improve the existing alpha-helix model and enable dynamics interactions.

## Data Availability

See section on Code Availability. RRID: SCR_019320.

## Code availability

All source code used to generate results, data and figures found within this manuscript are freely available within a GitHub repository under the Apache-2.0 licence. Additional information about the source code can be found in the repository. 10.5281/zenodo.4436309. https://github.com/GuyButcher/alpha-helix-coordinate-frame-simulation.
